# Supplementary material for: Under his thumb the effect of president Donald Trump’s Twitter messages on the US stock market
Source: PLoS One. 2020 Mar 11;15(3):e0229931. doi: 10.1371/journal.pone.0229931 (PMC7065837; doi:10.1371/journal.pone.0229931)
Supplement: S1 File — (DOCX) [file pone.0229931.s001.docx]

Under His Thumb

The Effect of President Donald Trump’s Twitter Messages on the US Stock Market

Supplementary Material

### S.A: SentiStrength

SentiStrength estimates the strength of positive and negative sentiment in short texts, even for informal language. It has human-level accuracy for short social web texts in English, except political texts. SentiStrength reports two sentiment strengths:

-1 (not negative) to -5 (extremely negative)

1 (not positive) to 5 (extremely positive)

It uses two scores because people process positive and negative sentiment in parallel - hence mixed emotions. SentiStrength can also report binary (positive/negative), trinary (positive/negative/neutral) and single scale (-4 to +4) results. SentiStrength was originally developed for English and optimised for general short social web texts but can be configured for other languages and contexts by changing its input files. Thus, SentiStrength is a sentiment analysis (opinion mining) program

To assess the accuracy of SentiStrength on a set of texts, a sample must first be classified and formatted. The human classifications can then be compared with the SentiStrength classifications on the same sample. Alternatively, if one data set is available to optimise the word strength list and the same set is to be used for validation then an alternative 10-fold cross-validation procedure can be used. This uses 90% of the data to train the term weights and the remaining 10% to assess the accuracy of the adjusted weights. This is repeated 10 times with a different 10% left out and the total results are reported.

An example how the SentiStrength tool gives the tweets a sentimental strength is as follows.

Tweet number 62 including the company American Airlines is used for the example:

*‘Thank you to Doug Parker and American Airlines for all of the help you have given to the U.S. with Hurricane flights. Fantastic job!’*

The whole tweet is imported to the SentiStrength algorithm and the output the algorithm gives is the following:

*‘Thank [+2] you to Doug Parker [proper noun] and American [proper noun] Airlines [proper noun] for all of the help you have given to the U.S. with Hurricane [proper noun] flights [-2]. Fantastic [+3] job! [+1 punctuation emphasis]’.*

In this case, the overall positive strength is +5 (since this is the maximum) and the negative strength is -2 which gives an overall strength of +3.

Distribution of SentiStrength sample scores (horizontal axis shows the frequency, vertical axis has the SentiStrength score):

S.B: Sensitivity analysis of tweets with positive sentiment vis-à-vis all tweets

| Day | AAR tweets with positive sentiment | p-value parametric test | p-value non-parametric test | AAR all tweets | p-value parametric test | p-value non-parametric test | Difference between tweets with positive sentiment and all tweets | p-value |
| --- | --- | --- | --- | --- | --- | --- | --- | --- |
| 0 | 0.0035 | 0.0830 | 0.0523 | 0.0010 | 0.5383 | 0.5636 | 0.0025 | 0.2114 |
| 1 | 0.0028 | 0.1346 | 0.1502 | -0.0007 | 0.6544 | 0.5636 | 0.0035 | 0.1891 |

S.C: Sensitivity analysis of tweets with negative sentiment vis-à-vis all tweets.

| Day | AAR tweets with negative sentiment | p-value parametric test | p-value non-parametric test | AAR all tweets | p-value parametric test | p-value non-parametric test | Difference between tweets with negative sentiment and all tweets | p-value |
| --- | --- | --- | --- | --- | --- | --- | --- | --- |
| 0 | -0.0037 | 0.0781 | 0.0905 | 0.0010 | 0.5383 | 0.5636 | 0.0048 | 0.0749 |
| 1 | -0.0071 | 0.0046 | 0.0905 | -0.0007 | 0.6544 | 0.5636 | 0.0064 | 0.1213 |

S.D: Sensitivity analysis of very strong tweets vis-à-vis neutral and moderate tweets.

Sensitivity analysis of SentiStrength score very strong (-4,-3, 3, 4) vis-à-vis moderate and neutral (-1, 0, 1)

| Day | AAR sentistrength very strong | p-value parametric | p-value non parametric | AAR sentistrength score moderate and neutral | p-value parametric | Pvalue non parametric | Difference AAR very strong vs moderate and neutral | p-value |
| --- | --- | --- | --- | --- | --- | --- | --- | --- |
| 0 | 0.0012 | 0.4107 | 0.0212 | 0.0031 | 0.0794 | 0.0000 | -0.0095 | 0.0424 |
| 1 | 0.0023 | 0.3356 | 0.0212 | 0.0000 | 0.4944 | 0.0000 | 0.0034 | 0.2847 |

Sensitivity analysis of SentiStrength score strong (-4, -3, -2, 2, 3, 4) vis-à-vis neutral (0)

| Day | AAR sentistrength score strong | p-value parametric | p-value non parametric | AAR sentistrength score neutral | p-value parametric | Pvalue non parametric | Difference AAR strong vs neutral | p-value |
| --- | --- | --- | --- | --- | --- | --- | --- | --- |
| 0 | -0.0030 | 0.09725 | 0.0000 | 0.0040 | 0.0962 | 0.0004 | -0.0048 | 0.1990 |
| 1 | -0.0022 | 0.1638 | 0.0000 | 0.0036 | 0.1220 | 0.0004 | 0.0011 | 0.7234 |

S.E: Sensitivity analysis of strong positively articulated tweets vis-à-vis neutral tweets and strong negatively articulated tweets in relation to neutral tweets.

Sensitivity analysis of SentiStrength score strong positive (2, 3, 4) vis-a-vis neutral (0)

| Day | Difference AAR strong positive  (2, 3, 4) vs neutral (0) | p-value |
| --- | --- | --- |
| 0 | 0.0002 | 0.9649 |
| 1 | 0.0039 | 0.4542 |

Sensitivity analysis of SentiStrength score strong negative (-2,-3,-4) vis-a-vis neutral (0)

| Day | Difference AAR strong negative  (-2,-3,-4) vs neutral (0) | p-value |
| --- | --- | --- |
| 0 | 0.0146 | 0.0134 |
| 1 | 0.0079 | 0.1083 |

Sensitivity analysis of SentiStrength score strong negative (-2,-3,-4) vis-a-vis strong positive (2, 3, 4)

| Day | Difference AAR strong negative  (-2,-3,-4) vs strong positive  (2, 3, 4) | p-value |
| --- | --- | --- |
| 0 | -0.0144 | 0.0257 |
| 1 | -0.0042 | 0.9474 |

## S.F: Event List

| **Event Number** | **Company name** | **Date** | **Time (EST)** | **Tweet** | **Opening/Closing** | **SentiStrength** | **Code** |
| --- | --- | --- | --- | --- | --- | --- | --- |
| 1 | Ford Motor | 17-11-16 | 21:01 | Just got a call from my friend Bill Ford Chairman of Ford who advised me that he will be keeping the Lincoln plant in Kentucky - no Mexico | opening | +1 | +1 |
|  |  |  | 21:15 | I worked hard with Bill Ford to keep the Lincoln plant in Kentucky. I owed it to the great State of Kentucky for their confidence in me! |  | +2 |  |
| 2 | United Technologies | 24-11-16 | 7:11 | I am working hard even on Thanksgiving trying to get Carrier A.C. Company to stay in the U.S. (Indiana). MAKING PROGRESS - Will know soon! | opening | 0 | 0 |
| 3 | United Technologies | 29-11-16 | 21:38 | I will be going to Indiana on Thursday to make a major announcement concerning Carrier A.C. staying in Indianapolis. Great deal for workers! | opening | +1 | +1 |
|  |  |  | 22:40 | Big day on Thursday for Indiana and the great workers of that wonderful state. We will keep our companies and jobs in the U.S. Thanks Carrier |  | +2 |  |
|  |  |  | 22:48 | Look forward to going to Indiana tomorrow in order to be with the great workers of Carrier. They will sell many air conditioners! |  | +2 |  |
|  |  |  | 22:50 | Getting ready to leave for the Great State of Indiana and meet the hard working and wonderful people of Carrier A.C. |  | +2 |  |
| 4 | Rexnord | 2-12-16 | 22:06 | Rexnord of Indiana is moving to Mexico and rather viciously firing all of its 300 workers. This is happening all over our country. No more! | opening | -3 | -1 |
| 5 | Boeing | 6-12-16 | 8:52 | Boeing is building a brand new 747 Air Force One for future presidents but costs are out of control more than $4 billion. Cancel order! | opening | -1 | -1 |
| 6 | Softbank | 6-12-16 | 14:10 | Masa said he would never do this had we (Trump) not won the election! | Closing | 0 | 0 |
| 7 | ExxonMobil | 11-12-16 | 10:29 | Whether I choose him or not for "State"- Rex Tillerson the Chairman &amp; CEO of ExxonMobil is a world class player and dealmaker. Stay tuned! | Closing | 0 | 0 |
| 8 | Lockheed Martin | 12-12-16 | 8:26 | The F-35 program and cost is out of control. Billions of dollars can and will be saved on military (and other) purchases after January 20th. | Opening | -1 | -1 |
| 9 | Lockheed Martin | 22-12-16 | 17:26 | Based on the tremendous cost and cost overruns of the Lockheed Martin F-35 I have asked Boeing to price-out a comparable F-18 Super Hornet! | Opening | +2 | +1 |
| 10 | Boeing | 22-12-16 | 17:26 | Based on the tremendous cost and cost overruns of the Lockheed Martin F-35 I have asked Boeing to price-out a comparable F-18 Super Hornet! | Opening | +2 | +1 |
| 11 | General Motors | 3-1-17 | 7:30 | General Motors is sending Mexican made model of Chevy Cruze to U.S. car dealers-tax free across border. Make in U.S.A. or pay big border tax! | Opening | -1 | -1 |
| 12 | Softbank | 3-1-17 | 13:44 | Trump is already delivering the jobs he promised America https://t.co/11spTMa6Tm | Closing | +1 | +1 |
| 13 | Ford Motor | 4-1-17 | 8:19 | Thank you to Ford for scrapping a new plant in Mexico and creating 700 new jobs in the U.S. This is just the beginning - much more to follow | Opening | +1 | +1 |
| 14 | Toyota Motor | 5-1-17 | 13:14 | Toyota Motor said will build a new plant in Baja Mexico to build Corolla cars for U.S. NO WAY! Build plant in U.S. or pay big border tax. | Closing | -1 | -1 |
| 15 | Fiat Chrysler | 9-1-17 | 9:14 | It's finally happening - Fiat Chrysler just announced plans to invest $1BILLION in Michigan and Ohio plants adding 2000 jobs. This after... | Opening | 0 | +1 |
|  |  |  | 9:16 | Ford said last week that it will expand in Michigan and U.S. instead of building a BILLION dollar plant in Mexico. Thank you Ford &amp; Fiat C! |  | +1 |  |
| 16 | Ford | 9-1-17 | 9:14 | It's finally happening - Fiat Chrysler just announced plans to invest $1BILLION in Michigan and Ohio plants adding 2000 jobs. This after... | Opening | 0 | +1 |
|  |  |  | 9:16 | Ford said last week that it will expand in Michigan and U.S. instead of building a BILLION dollar plant in Mexico. Thank you Ford &amp; Fiat C! |  | +1 |  |
| 17 | General Motors | 17-1-17 | 12:55 | Thank you to General Motors and Walmart for starting the big jobs push back into the U.S.! | Closing | +1 | +1 |
| 18 | Walmart | 17-1-17 | 12:55 | Thank you to General Motors and Walmart for starting the big jobs push back into the U.S.! | Closing | +1 | +1 |
| 19 | Lockheed Martin | 18-1-17 | 7:34 | Totally biased @NBCNews went out of its way to say that the big announcement from Ford G.M. Lockheed &amp; others that jobs are coming back... … to the U.S., but had nothing to do with TRUMP, is more FAKE NEWS. Ask top CEO’s of those companies for real facts. Came back because of me! | Opening | -1 | -1 |
| 20 | Ford Motor | 18-1-17 | 7:34 | Totally biased @NBCNews went out of its way to say that the big announcement from Ford G.M. Lockheed &amp; others that jobs are coming back... … to the U.S., but had nothing to do with TRUMP, is more FAKE NEWS. Ask top CEO’s of those companies for real facts. Came back because of me! | Opening | -1 | -1 |
| 21 | Bayer AG | 18-1-17 | 8:00 | Bayer AG has pledged to add U.S. jobs and investments after meeting with President-elect Donald Trump the latest in a string... @WSJ | Opening | 0 | 0 |
| 22 | General Motor | 24-1-17 | 14:46 | Great meeting with Ford CEO Mark Fields and General Motors CEO Mary Barra at the @WhiteHouse today. https://t.co/T0eIgO6LP8 | Closing | +2 | +1 |
| 23 | Ford Motor | 24-1-17 | 14:46 | Great meeting with Ford CEO Mark Fields and General Motors CEO Mary Barra at the @WhiteHouse today. https://t.co/T0eIgO6LP8 | Closing | +2 | +1 |
| 24 | Delta Air Lines | 30-1-17 | 7:16 | Only 109 people out of 325,000 were detained and held for questioning. Big problems at airports were caused by Delta computer outage,..... | Opening | -1 | -1 |
| 25 | Samsung Electronics | 2-2-17 | 12:29 | Thank you @Samsung! We would love to have you! https://t.co/r5nxC9oOA4 | Closing | +1 | +1 |
| 26 | Nordstrom | 8-2-17 | 10:51 | My daughter Ivanka has been treated so unfairly by @Nordstrom. She is a great person -- always pushing me to do the right thing! Terrible! | Closing | -1 | -1 |
| 27 | Intel | 08-02-17 | 14:22 | Thank you Brian Krzanich CEO of @Intel. A great investment ($7 BILLION) in American INNOVATION and JOBS!… https://t.co/oicfDsPKHQ | Closing | +2 | +1 |
| 28 | Lockheed Martin | 11-02-17 | 8:24 | I am reading that the great border WALL will cost more than the government originally thought but I have not gotten involved in the........design or negotiations yet. When I do just like with the F-35 FighterJet or the Air Force One Program price will come WAY DOWN! | Opening | 0 | 0 |
| 29 | Humana | 14-2-17 | 17:50 | Obamacare continues to fail. Humana to pull out in 2018. Will repeal replace &amp; save healthcare for ALL Americans. https://t.co/glWEQ0lNR4 | Opening | -1 | -1 |
| 30 | Aetna | 15-2-17 | 16:34 | Aetna CEO: Obamacare in 'Death Spiral' #RepealAndReplace https://t.co/dmHL7xIEQv | Opening | 0 | 0 |
| 31 | Boeing | 17-2-17 | 6:38 | Going to Charleston South Carolina in order to spend time with Boeing and talk jobs! Look forward to it. | Opening | 0 | 0 |
| 32 | Exxon Mobil | 6-2-17 | 16:19 | 'President Trump Congratulates Exxon Mobil for Job-Creating Investment Program'https://t.co/adBzWhtq8S | Opening | +1 | +1 |
|  |  | 6-2-17 | 16:21 | Buy American &amp; hire American are the principals at the core of my agenda which is: JOBS JOBS JOBS! Thank you @exxonmobil. |  | +1 |  |
|  |  | 6-2-17 | 22:49 | Buy American &amp; hire American are the principles at the core of my agenda which is: JOBS JOBS JOBS! Thank you @exxonmobil. |  | +1 |  |
|  |  | 6-2-17 | 22:50 | Thank you to @exxonmobil for your $20 billion investment that is creating more than 45000 manufacturing &amp; construction jobs in the USA! |  | +1 |  |
| 33 | Transcanada | 24-3-17 | 12:03 | Today I was pleased to announce the official approval of the presidential permit for the #KeystonePipeline. A grea… https://t.co/GWNo2XAueg | Closing | +1 | +1 |
| 34 | Charter Communications inc | 24-3-17 | 12:59 | Today I was thrilled to announce a commitment of $25 BILLION &amp; 20K AMERICAN JOBS over the next 4 years. THANK YOU… https://t.co/nWJ1hNmzoR | Closing | +3 | +1 |
| 35 | Ford Motor | 28-3-17 | 6:36 | Big announcement by Ford today. Major investment to be made in three Michigan plants. Car companies coming back to U.S. JOBS! JOBS! JOBS! | Opening | 0 | 0 |
| 36 | General Motor | 5-4-17 | 22:12 | JOBS JOBS JOBS!https://t.co/XGOQPHywrt https://t.co/B5Qbn6llzE | Opening | +1 | +1 |
| 37 | Ford Motor | 5-4-17 | 22:12 | JOBS JOBS JOBS!https://t.co/XGOQPHywrt https://t.co/B5Qbn6llzE | Opening | +1 | +1 |
| 38 | Walmart | 5-4-17 | 22:12 | JOBS JOBS JOBS!https://t.co/XGOQPHywrt https://t.co/B5Qbn6llzE | Opening | +1 | +1 |
| 39 | Sprint | 5-4-17 | 22:12 | JOBS JOBS JOBS!https://t.co/XGOQPHywrt https://t.co/B5Qbn6llzE | Opening | +1 | +1 |
| 40 | Kroger | 5-4-17 | 22:12 | JOBS JOBS JOBS!https://t.co/XGOQPHywrt https://t.co/B5Qbn6llzE | Opening | +1 | +1 |
| 41 | Fiat Chrysler | 5-4-17 | 22:12 | JOBS JOBS JOBS!https://t.co/XGOQPHywrt https://t.co/B5Qbn6llzE | Opening | +1 | +1 |
| 42 | Alibaba | 5-4-17 | 22:12 | JOBS JOBS JOBS!https://t.co/XGOQPHywrt https://t.co/B5Qbn6llzE | Opening | +1 | +1 |
| 43 | Amazon | 5-4-17 | 22:12 | JOBS JOBS JOBS!https://t.co/XGOQPHywrt https://t.co/B5Qbn6llzE | Opening | +1 | +1 |
| 44 | Aetna | 4-5-17 | 7:38 | Death spiral!'Aetna will exit Obamacare markets in VA in 2018 citing expected losses on INDV plans this year'https://t.co/5YnzDitF8r | Opening | -2 | -1 |
| 45 | Rexnord | 8-5-17 | 17:58 | Rexnord of Indiana made a deal during the Obama Administration to move to Mexico. Fired their employees. Tax product big that's sold in U.S. | Opening | -2 | -1 |
| 46 | Facebook | 1-6-17 | 20:40 | Crooked Hillary Clinton now blames everybody but herself refuses to say she was a terrible candidate. Hits Facebook &amp; even Dems &amp; DNC. | Opening | -3 | -1 |
| 47 | Goldman Sachs | 9-6-17 | 10:22 | Congratulations to Jeb Hensarling & Republicans on successful House vote to repeal major parts of the 2010 Dodd-Frank financial law. GROWTH! | Closing | +1 | +1 |
| 48 | Morgan Stanley | 9-6-17 | 10:22 | Congratulations to Jeb Hensarling & Republicans on successful House vote to repeal major parts of the 2010 Dodd-Frank financial law. GROWTH! | Closing | +1 | +1 |
| 49 | Amazon | 28-6-17 | 8:06 | The #AmazonWashingtonPost sometimes referred to as the guardian of Amazon not paying internet taxes (which they should) is FAKE NEWS! | Opening | -1 | -1 |
| 50 | Pfizer | 21-7-17 | 23:31 | Billions of dollars in investments &amp; thousands of new jobs in America! An initiative via Corning Merck &amp; Pfizer: https://t.co/QneN48bSiq https://t.co/5VtMfuY3PM | Opening | 0 | 0 |
| 51 | Merck | 21-7-17 | 23:31 | Billions of dollars in investments &amp; thousands of new jobs in America! An initiative via Corning Merck &amp; Pfizer: https://t.co/QneN48bSiq https://t.co/5VtMfuY3PM | Opening | 0 | 0 |
| 52 | Corning | 21-7-17 | 23:31 | Billions of dollars in investments &amp; thousands of new jobs in America! An initiative via Corning Merck &amp; Pfizer: https://t.co/QneN48bSiq https://t.co/5VtMfuY3PM | Opening | 0 | 0 |
| 53 | Amazon | 22-7-17 | 6:33 | A new INTELLIGENCE LEAK from the Amazon Washington Post this time against A.G. Jeff Sessions.These illegal leaks like Comey's must stop! | Opening | 0 | -1 |
|  |  | 23-7-17 | 18:57 | It's hard to read the Failing New York Times or the Amazon Washington Post because every story/opinion even if should be positive is bad! |  | 0 |  |
|  |  | 24-7-17 | 22:23 | The Amazon Washington Post fabricated the facts on my ending massive dangerous and wasteful payments to Syrian rebels fighting Assad..... |  | -2 |  |
|  |  | 24-7-17 | 22:28 | So many stories about me in the @washingtonpost are Fake News. They are as bad as ratings challenged @CNN. Lobbyist for Amazon and taxes? |  | -1 |  |
|  |  | 24-7-17 | 22:36 | Is Fake News Washington Post being used as a lobbyist weapon against Congress to keep Politicians from looking into Amazon no-tax monopoly? |  | -1 |  |
| 54 | Amazon | 8-8-17 | 13:00 | E-mails show that the AmazonWashingtonPost and the FailingNewYorkTimes were reluctant to cover the Clinton/Lynch secret meeting in plane. | Closing | -2 | -1 |
| 55 | Merck | 14-8-17 | 8:54 | Now that Ken Frazier of Merck Pharma has resigned from President's Manufacturing Councilhe will have more time to LOWER RIPOFF DRUG PRICES! | Opening | -1 | -1 |
|  |  |  | 18:09 | .@Merck Pharma is a leader in higher &amp; higher drug prices while at the same time taking jobs out of the U.S. Bring jobs back &amp; LOWER PRICES! |  | 0 |  |
| 56 | Amazon | 16-8-17 | 6:12 | Amazon is doing great damage to tax paying retailers. Towns cities and states throughout the U.S. are being hurt - many jobs being lost! | Opening | 0 | 0 |
| 57 | Facebook | 23-8-17 | 7:18 | Thank you Arizona. Beautiful turnout of 15000 in Phoenix tonight! Full coverage of rally via my Facebook at: https://t.co/s0D12EFs40 https://t.co/WT4D9Vsen1 | Opening | +2 | +1 |
| 58 | Andeavor | 6-9-17 | 19:20 | Wonderful to be in North Dakota with the incredible hardworking men &amp; women @ the Andeavor Refinery. Full remarks: ??https://t.co/uxBpyeERUm https://t.co/fV9R9gJxDS | Opening | +2 | +1 |
| 59 | Twitter | 11-9-17 | 11:41 | FLORIDA- Visit https://t.co/pdBaD9t8SK to find shelters road closures &amp; evacuation routes. Helpful Twitter list: https://t.co/BUMVlxGFe8 https://t.co/ncnuqCeB5K | Closing | +1 | +1 |
| 60 | Disney | 15-9-17 | 7:20 | ESPN is paying a really big price for its politics (and bad programming). People are dumping it in RECORD numbers. Apologize for untruth! | Opening | -1 | -1 |
| 61 | Facebook | 22-9-17 | 6:44 | The Russia hoax continues now it's ads on Facebook. What about the totally biased and dishonest Media coverage in favor of Crooked Hillary? | Opening | 0 | 0 |
| 62 | American Airline | 22-9-17 | 13:54 | Thank you to Doug Parker and American Airlines for all of the help you have given to the U.S. with Hurricane flights. Fantastic job! | Closing | +3 | +1 |
| 63 | Facebook | 27-9-17 | 8:36 | Facebook was always anti-Trump.The Networks were always anti-Trump henceFake News @nytimes(apologized) &amp; @WaPo were anti-Trump. Collusion? | Opening | -1 | -1 |
| 64 | Facebook | 23-10-17 | 16:06 | Keep hearing about "tiny" amount of money spent on Facebook ads. What about the billions of dollars of Fake News on CNN ABC NBC &amp; CBS? | Closing | -1 | -1 |
| 65 | Broadcom | 2-11-17 | 15:58 | Today we are thrilled to welcome @Broadcom CEO Hock Tan to the WH to announce he is moving their HQ’s from Singapore back to the U.S.A..... https://t.co/WrqUXBndyZ | Closing | +3 | +1 |
|  |  |  | 16:33 | Broadcom's move to America=$20 BILLION of annual rev into U.S.A. $3+ BILLION/yr. in research/engineering &amp; $6 BILLION/yr. in manufacturing. https://t.co/NsJ4PtVTtl |  | 0 |  |
| 66 | Twitter | 3-11-17 | 6:51 | My Twitter account was taken down for 11 minutes by a rogue employee. I guess the word must finally be getting out-and having an impact. | Opening | -1 | -1 |
| 67 | Wells Fargo | 8-12-17 | 10:18 | Fines and penalties against Wells Fargo Bank for their bad acts against their customers and others will not be dropped as has incorrectly been reported but will be pursued and if anything substantially increased. I will cut Regs but make penalties severe when caught cheating! | Closing | -2 | -1 |
| 68 | Amazon | 29-12-17 | 8:04 | Why is the United States Post Office which is losing many billions of dollars a year while charging Amazon and others so little to deliver their packages making Amazon richer and the Post Office dumber and poorer? Should be charging MUCH MORE! | Opening | -2 | -1 |
| 69 | Apple | 18-1-18 | 18:28 | I promised that my policies would allow companies like Apple to bring massive amounts of money back to the United States. Great to see Apple follow through as a result of TAX CUTS. Huge win for American workers and the USA! https://t.co/OwXVUyLOb1 | Opening | +1 | +1 |
| 70 | JP Morgan Chase | 24-1-18 | 6:58 | Tremendous investment by companies from all over the world being made in America. There has never been anything like it. Now Disney J.P. Morgan Chase and many others. Massive Regulation Reduction and Tax Cuts are making us a powerhouse again. Long way to go! Jobs Jobs Jobs! | Opening | 0 | 0 |
| 71 | Disney | 24-1-18 | 3:58 | Tremendous investment by companies from all over the world being made in America. There has never been anything like it. Now Disney J.P. Morgan Chase and many others. Massive Regulation Reduction and Tax Cuts are making us a powerhouse again. Long way to go! Jobs Jobs Jobs! | Opening | 0 | 0 |
| 72 | Facebook | 17-2-18 | 15:11 | The Fake News Media never fails. Hard to ignore this fact from the Vice President of Facebook Ads Rob Goldman! https://t.co/XGC7ynZwYJ | Closing | +1 | +1 |
|  |  | 17-2-18 | 15:16 | “I have seen all of the Russian ads and I can say very definitively that swaying the election was *NOT* the main goal.”Rob GoldmanVice President of Facebook Ads https://t.co/A5ft7cGJkE |  | 0 |  |
| 73 | Amazon | 29-3-18 | 7:57 | I have stated my concerns with Amazon long before the Election. Unlike others they pay little or no taxes to state &amp; local governments use our Postal System as their Delivery Boy (causing tremendous loss to the U.S.) and are putting many thousands of retailers out of business! | Opening | -2 | -1 |
| 74 | Amazon | 31-3-18 | 8:45 | While we are on the subject it is reported that the U.S. Post Office will lose $1.50 on average for each package it delivers for Amazon. That amounts to Billions of Dollars. The Failing N.Y. Times reports that “the size of the company’s lobbying staff has ballooned” and that... | Opening | -2 | -1 |
|  |  |  | 8:52 | ...does not include the Fake Washington Post which is used as a “lobbyist” and should so REGISTER. If the P.O. “increased its parcel rates Amazon’s shipping costs would rise by $2.6 Billion.” This Post Office scam must stop. Amazon must pay real costs (and taxes) now! |  | -1 |  |
|  |  | 2-4-18 | 9:35 | Only fools or worse are saying that our money losing Post Office makes money with Amazon. THEY LOSE A FORTUNE and this will be changed. Also our fully tax paying retailers are closing stores all over the country...not a level playing field! |  | -1 |  |
|  |  |  | 9:55 | I am right about Amazon costing the United States Post Office massive amounts of money for being their Delivery Boy. Amazon should pay these costs (plus) and not have them bourne by the American Taxpayer. Many billions of dollars. P.O. leaders don’t have a clue (or do they?)! |  | 0 |  |
| 75 | Amazon | 5-4-18 | 9:10 | The Fake News Washington Post Amazon’s “chief lobbyist” has another (of many) phony headlines “Trump Defiant As China Adds Trade Penalties.” WRONG! Should read “Trump Defiant as U.S. Adds Trade Penalties Will End Barriers And Massive I.P. Theft.” Typically bad reporting! | Opening | -1 | -1 |
| 76 | Soutwest Airlines | 1-5-18 | 15:42 | Today it was my great honor to thank and welcome heroic crew members and passengers of Southwest Airlines Flight 1380 at the @WhiteHouse! https://t.co/fYYgWToddi | Closing | +2 | +1 |
| 77 | AT&T | 14-5-18 | 19:49 | Why doesn’t the Fake News Media state that the Trump Administration’s Anti-Trust Division has been and is opposed to the AT&amp;T purchase of Time Warner in a currently ongoing Trial. Such a disgrace in reporting! | Opening | -2 | -1 |
| 78 | Harley Davidson | 25-6-18 | 17:28 | Surprised that Harley-Davidson of all companies would be the first to wave the White Flag. I fought hard for them and ultimately they will not pay tariffs selling into the E.U. which has hurt us badly on trade down $151 Billion. Taxes just a Harley excuse - be patient! #MAGA | Opening | -2 | -1 |
|  |  | 26-6-18 | 7:16 | Early this year Harley-Davidson said they would move much of their plant operations in Kansas City to Thailand. That was long before Tariffs were announced. Hence they were just using Tariffs/Trade War as an excuse. Shows how unbalanced &amp; unfair trade is but we will fix it..... |  | -2 |  |
|  |  |  | 7:25 | ....We are getting other countries to reduce and eliminate tariffs and trade barriers that have been unfairly used for years against our farmers workers and companies. We are opening up closed markets and expanding our footprint. They must play fair or they will pay tariffs! |  | -1 |  |
|  |  |  | 7:37 | ....When I had Harley-Davidson officials over to the White House I chided them about tariffs in other countries like India being too high. Companies are now coming back to America. Harley must know that they won’t be able to sell back into U.S. without paying a big tax! |  | 0 |  |
|  |  |  | 7:49 | ....We are finishing our study of Tariffs on cars from the E.U. in that they have long taken advantage of the U.S. in the form of Trade Barriers and Tariffs. In the end it will all even out - and it won’t take very long! |  | -1 |  |
|  |  |  | 8:17 | A Harley-Davidson should never be built in another country-never! Their employees and customers are already very angry at them. If they move watch it will be the beginning of the end - they surrendered they quit! The Aura will be gone and they will be taxed like never before! |  | -3 |  |
| 79 | Harley Davidson | 27-6-18 | 11:26 | Harley-Davidson should stay 100% in America with the people that got you your success. I’ve done so much for you and then this. Other companies are coming back where they belong! We won’t forget and neither will your customers or your now very HAPPY competitors! | Closing | +2 | N/A |
| 80 | Harley Davidson | 3-7-18 | 10:00 | Now that Harley-Davidson is moving part of its operation out of the U.S. my Administration is working with other Motor Cycle companies who want to move into the U.S. Harley customers are not happy with their move - sales are down 7% in 2017. The U.S. is where the Action is! | Closing | -1 | -1 |
| 81 | Twitter | 7-7-18 | 9:21 | Twitter is getting rid of fake accounts at a record pace. Will that include the Failing New York Times and propaganda machine for Amazon the Washington Post who constantly quote anonymous sources that in my opinion don’t exist - They will both be out of business in 7 years! | Opening | -2 | -1 |
| 82 | Pfizer | 9-7-18 | 13:08 | Pfizer &amp; others should be ashamed that they have raised drug prices for no reason. They are merely taking advantage of the poor &amp; others unable to defend themselves while at the same time giving bargain basement prices to other countries in Europe &amp; elsewhere. We will respond! | Closing | -1 | +1 |
|  |  |  | 17:37 | Just talked with Pfizer CEO and @SecAzar on our drug pricing blueprint. Pfizer is rolling back price hikes so American patients don’t pay more. We applaud Pfizer for this decision and hope other companies do the same. Great news for the American people! |  | +2 |  |
| 83 | Pfizer | 19-7-18 | 6:23 | Thank you to Novartis for not increasing your prices on prescription drugs. Likewise to Pfizer. We are making a big push to actually reduce the prices maybe substantially on prescription drugs. | Opening | +1 | +1 |
| 84 | Novartis | 19-7-18 | 6:23 | Thank you to Novartis for not increasing your prices on prescription drugs. Likewise to Pfizer. We are making a big push to actually reduce the prices maybe substantially on prescription drugs. | Opening | +1 | +1 |
| 85 | Amazon | 23-7-18 | 9:21 | The Amazon Washington Post has gone crazy against me ever since they lost the Internet Tax Case in the U.S. Supreme Court two months ago. Next up is the U.S. Post Office which they use at a fraction of real cost as their “delivery boy” for a BIG percentage of their packages.... | Opening | +1 | 0 |
|  |  |  | 9:35 | ....In my opinion the Washington Post is nothing more than an expensive (the paper loses a fortune) lobbyist for Amazon. Is it used as protection against antitrust claims which many feel should be brought? |  | -1 |  |
| 86 | Ford Motor | 26-7-18 | 17:45 | Sergio Marchionne who passed away today was one of the most brilliant &amp; successful car executives since the days of the legendary Henry Ford. It was a great honor for me to get to know Sergio as POTUS he loved the car industry and fought hard for it. He will be truly missed! | Opening | +1 | +1 |
| 87 | Twitter | 26-7-18 | 7:46 | Twitter “SHADOW BANNING” prominent Republicans. Not good. We will look into this discriminatory and illegal practice at once! Many complaints. | Opening | -1 | -1 |
| 88 | Amazon | 30-7-18 | 15:09 | ...and the Amazon Washington Post do nothing but write bad stories even on very positive achievements - and they will never change! | Closing | +2 | +1 |
| 89 | Amazon | 2-8-18 | 6:38 | Congratulations to @GreggJarrett on The TREMENDOUS success of his just out book “The Russia Hoax The Illicit Scheme To Clear Hillary Clinton &amp; Frame Donald Trump.” Already number one on Amazon. Hard work from a brilliant guy. It’s the Real Story of the Rigged Witch Hunt! | Opening | +2 | +1 |
| 90 | Amazon | 6-8-18 | 23:01 | Congratulations to Gregg Jarrett on his book “THE RUSSIA HOAX THE ILLICIT SCHEME TO CLEAR HILLARY CLINTON AND FRAME DONALD TRUMP” going to #1 on @nytimes and Amazon. It is indeed a HOAX and WITCH HUNT illegally started by people who have already been disgraced. Great book! | Opening | -1 | -1 |
| 91 | Apple | 13-8-18 | 18:47 | Had a very good phone call with @EmmanuelMacron President of France. Discussed various subjects in particular Security and Trade. Many other calls and conversations today. Looking forward to dinner tonight with Tim Cook of Apple. He is investing big dollars in U.S.A. | Opening | +2 | +1 |
| 92 | Target | 24-8-18 | 5:57 | Target CEO raves about the Economy. “This is the best consumer environment I’ve seen in my career.” A big statement from a top executive. But virtually everybody is saying this, & when our Trade Deals are made, & cost cutting done, you haven’t seen anything yet! @DRUDGE_REPORT | Opening | +1 | +1 |
| 93 | Nike | 5-9-18 | 9:39 | Just like the NFL whose ratings have gone WAY DOWN Nike is getting absolutely killed with anger and boycotts. I wonder if they had any idea that it would be this way? As far as the NFL is concerned I just find it hard to watch and always will until they stand for the FLAG! | Closing | -4 | -1 |
| 94 | Nike | 7-9-18 | 6:56 | What was Nike thinking? | Opening | 0 | 0 |
| 95 | Apple | 8-9-18 | 11:45 | Apple prices may increase because of the massive Tariffs we may be imposing on China - but there is an easy solution where there would be ZERO tax and indeed a tax incentive. Make your products in the United States instead of China. Start building new plants now. Exciting! #MAGA | Closing | +1 | +1 |
| 96 | Ford Motor | 9-9-18 | 9:49 | “Ford has abruptly killed a plan to sell a Chinese-made small vehicle in the U.S. because of the prospect of higher U.S. Tariffs.” CNBC. This is just the beginning. This car can now be BUILT IN THE U.S.A. and Ford will pay no tariffs! | Closing | -3 | -1 |
| 97 | Facebook | 21-10-18 | 18:48 | Facebook has just stated that they are setting up a system to “purge” themselves of Fake News. Does that mean CNN will finally be put out of business? | Opening | -1 | -1 |
| 98 | Twitter | 26-10-18 | 10:15 | Twitter has removed many people from my account and, more importantly, they have seemingly done something that makes it much harder to join - they have stifled growth to a point where it is obvious to all. A few weeks ago it was a Rocket Ship, now it is a Blimp! Total Bias? | Closing | -1 | -1 |
| 99 | Wells Fargo | 30-10-18 | 8:53 | “If the Fed backs off and starts talking a little more Dovish, I think we’re going to be right back to our 2,800 to 2,900 target range that we’ve had for the S&P 500.” Scott Wren, Wells Fargo. | Opening | 0 | 0 |
| 100 | Facebook | 3-11-18 | 17:05 | Rumor has it that senator Joe Donnelly of Indiana is paying for Facebook ads for his so-called opponent on the libertarian ticket. Donnelly is trying to steal the election? Isn’t that what Russia did!? | Opening | -2 | -1 |

Overview of tweets posted by President Trump including a company name (columns 2 and 5). The date and time are in Eastern Time (columns 3 and 4). When the tweet is posted before the market opens the tweets get labeled as opening and otherwise closing label (column 6). The SentiStrength shows the degree of negative/positive sentimental value of the tweet ranging from -5 to +5(column 7). Column (8) shows the overall sentimental strength of the tweet +1 (positive), 0 (neutral), -1 negative (column 8).
