# Supplementary material for: Under his thumb the effect of president Donald Trump’s Twitter messages on the US stock market
Source: PLoS One. 2020 Mar 11;15(3):e0229931. doi: 10.1371/journal.pone.0229931 (PMC7065837; doi:10.1371/journal.pone.0229931)
Supplement: Attachment — Submitted filename: PONE-D-19-26269 Rebuttal.docx [file pone.0229931.s002.docx]

PONE-D-19-26269 Rebuttal

Dear Alexandre Bovet.

Thank you very much for giving us the opportunity to revise and resubmit our manuscript to PLOS ONE. We also want to thank the reviewers for taking the time to study our paper and to provide thoughtful and constructive suggestions. This is highly appreciated. We feel that addressing the issues raised by the reviewers has helped to improve the manuscript a lot. A detailed account regarding each point raised goes below. We very much do hope that you will feel that the revised manuscript meets the criteria for publication.

Yours sincerely, also on behalf of my co-author,

XXXXXXXXXXX

| # | Comment | Response^[1]^ |
| --- | --- | --- |
| Reviewer 1 | | |
| 1 | The authors investigate the effects of President Donald Trump's Twitter use on financial returns of companies he mentions at the daily timescale using an event study methodology. The analysis is conducted appropriately, however some clarifications are needed in order for the manuscript to be publishable in my opinion. | Thank you very much for taking the time to read our manuscript and provide suggestions. |
| 2 | The authors declare they eliminate from their analysis any tweet that was online for less than 24 hours. This seems to me an unnecessary restriction, since financial markets react on a much faster time scale nowadays and tweet deletion doesn't necessarily imply that it would have no effect. I invite the authors to expand on the justification for this choice of threshold; | Thanks for raising this issue. The decision to delete tweets that were online for less than 24 hours is based upon a limitation on the data archive. Unfortunately, TrumpTwitterarchive.com/archive has a breakpoint in their data series, from 27/1/2017 onwards the archive switched to monitoring the twitter tweets in real time. Before this date, the tweets were only collected daily (at least every 24 hours). Therefore, not all tweets that are deleted can be collected in the data archive. With certainty, it can be said that all the tweets that have been online for over 24 hours are included in the dataset and therefore the decision has been made to eliminate all tweets that are deleted within the time span of 24 hours to avoid a breakpoint in the dataset that is unrelated to the topic studied. {161-171} |
| 3 | The authors refer numerous times to the President's "temperamental nature". While it can be understood why they call it like that, it seems inappropriate to me on a scientific journal and would suggest they find an agreement with the editor on the choice of wording; | This point is well taken. We amend the choice of wording when we refer to the tweets of the US president. Reviewer 2 also raised this issue (see comment #19) {65-66, 196-197, 360} |
| 4 | I would suggest the authors introduce the event study methodology and statistical testing with more detail, giving particular attention to the distinction between quantities calculated in the estimation period or in the observation period. | We provide more detail about the methodology and the tests employed. We point out the importance of the estimation period for the calculations of the AARs in the event period. {143-156} |
| 5 | In the definition of the CDA test statistic, \overline{u} and \overline{u_it} appear in the formulas but are never defined. This makes the explanation particularly confusing.  I strongly suggest that the authors revise the whole section and spend more words explaining the methods, since they cannot be easily understood from the text as they are written; | We provide more detail about this test statistic. {245-257}  Thanks for suggesting, please see the changes in relation to the previous comment (#4). |
| 6 | Reference to the original paper by Cowan (Cowan, A. R. (1992). Nonparametric event study tests. Review of Quantitative Finance and Accounting, 2(4), 343-358) should be there; | Thanks for suggesting this; we include it in the list of references. {261, 432-433} |
| 7 | In the definition of the non-parametric test, no clear definition is given for quantities M_i and S_it. Also, there is some confusing phrasing regarding positive abnormal returns (line 224) and negative abnormal returns (line 227), which should be made more clear. | We amended the text; see also response regarding comment #5., We provide more detail. {245-257, 259-267} |
| 8 | In Table 1, a p-value for the Jarque-Bera test would be easier to understand for readers who don't know the quantiles of the Chi Squared distribution by heart. | We include the p-value. {307; last line in the table} |
| 9 | Figure 1 panels should be made of the same size and with same x axis limits in order to be easier to read and compare. Also, axis labels instead of plot titles would make the plot easier to understand at a glance. | Thanks for suggesting this; we changed the formatting and included the axis labels. {322-326} |
| Reviewer 2 | | |
| 10 | The paper presents an analysis of the effects of president Trump twitter messages on the stock market. The paper is rigorous in the data analysis and comply with the strict requirements of PLOS ONE with regards to data availability and statistical analysis. However, I have a number of complaints about the data used in the paper, the presentation and justification of the data analysis techniques employed and the strength and interpretation of the conclusions. I think these complaints should be acted upon to make the paper better. | We very much appreciate you took the time to read our manuscript and to provide comments. |
| 11 | The dataset used in the analysis, in my view, is too restrictive. There is no really justification on the reasons it should be based just on the first two years of presidency of Trump. I can understand the reasons of the starting date, but there is no real reason to restrict it to just two years. In fact, it would have been much more useful to extend it to the present day (so one additional year) and then study if the activity of the president actually made the found effects on the market and perhaps showed some decay over time. The major problem is that the data set is too small, once irrelevant or questionable tweets are removed. The analysis is based on only 100 tweets! Not to mention that these 100 tweets then become only 81 once sentiment analysis is applied to them, (assuming the 19 neutral tweets did not find any use)! | This is a comment that is highly relevant in the light of conducting an event study; The issue of how many events are ‘enough’ is heavily debated. There are a large number of event studies that rely on less than 10 events and event studies which have one event only.  First, all tweets were checked for company names; this relates to about 7000 tweets per year. Companies are not mentioned that often and we selected all of them as described in the manuscript {158-180}. Compared to existing research, it shows that the related studies have much less tweets in the sample than we do, namely Born et al. – 15; Ge et al. – 48; Juma’h and Alnsour – 58.  Second, to substantiate that our sample size is not overly restrictive, we refer to MacKinlay (1997; table 2, page 97) and the simulations by Brown and Warner (1985). This suggests that our sample size is not too small from an event study perspective.  Last, an event study is not very suitable to investigate long-time effects of news. Most importantly, this is because of the continuous build-up of confounding events (news) that happens after the original event. |
| 12 | The set is too small, in my view, to support the analysis that followed as other factors happening at the same time could have produced the observed result. The use of more data would certainly make this possibility smaller. | See also our response to the previous comment. We control for confounding news regarding the companies as explained in the data section. {171-174} |
| 13 | For the same reasons I would also extend the event window from 2 to maybe 3 or 4 days. This would enable to account for holidays or other delaying factors on the effect the tweet could have on the market. Also, an analysis of the effects of the window size on the conclusions would also be quite interesting. | Thanks for raising this important issue. We account for holidays and weekends by using trading days throughout the analysis. We realize this might not have been very clear and now explain it in the text. {207-209}  Further expanding the event window conflicts with the assumptions of relying on the market and risk adjusted returns model and would reduce the power of all test stats. The assumption is that market participants can respond quickly to news and that their combined efforts reflect in the stock prices (returns). Changing to a long event window reduces the power of the tests and opens up the impact of more and more confounding events (news). In addition, it would rely on the assumption that investors would start to respond to a tweet after some days. This is not in line with finance theory though. |
| 14 | the distinction between "estimation window" and "event window" is not clear. | Thank you for highlighting this; Reviewer 1 also raises the point (comment #4). We now understand the need to explain the research method and provide a more detailed explanation of the event study method and point out the differences between the two windows. {143-156} |
| 15 | Why did you not had access to more high-frequency information? One of the authors is from a school of management and so this should not have been so difficult. | Unfortunately, our university is not endowed well and is not willing to fund access to the databases that include HF information / tick data. |
| 16 | While the formulation of the hypnotises and style of the analysis is very clear and presented with rigour, the motivations for the choice of some of the techniques has not been clearly justified. Some more explanation on the reasons you chose the "market and risk adjusted returns model" would have been useful. More importantly, why did you choose the "crude dependence adjustment model"? And could you provide some reference for it? Why did you use the Jarque-Bera statistics? Why not one of the many other statistics? The choice of some of these methods should be clearly justified so as not to suggest the choice could have been biased by the results. | See also the response to comments #12-14. We provide more detail about the event study methodology and motivate the use of the market and risk adjusted model, CDA, and provide reference. {143-156, 241-271, 432-433}  We use the JB to inform about the (non)normality of the distribution. We do perform both parametric and non-parametric testing to rule out bias. |
| 17 | what does the index t stands for in the formula at page 8 line 208? | The ‘t’ is for the day in the event window. We now also mention this in the accompanying text. {248} |
| 18 | Why loosing the strength of the sentiment provided by SentiStrength and just accounting for its positive and negative nature (as reported in line 244, page 10)? It would have been quite interesting to relate the strength of the opinion to its effects. It could have provided also more interesting arguments for the analysis reported at the end of page 14. | Thank you very much for suggesting this. It is very useful. We include an assessment of whether the strength of sentiment matters. We study whether (very) ‘strong’ expressions yield abnormal returns that significantly deviate from moderate/neutral. We study whether ‘strong’ positive yields a different response than ‘strong’ negative responses. {375-384; see also Supplementary Material D and E} |
| 19 | I fund quite strange that in a scientific paper the authors could make such "personal" and "opinionated" comments such as: "We assume that the temperamental nature of the president results in lack of predictability" (page 3, line 64) or again "temperamental nature" (page 7, line 163) or, finally, your mention of the possibility that someone with prior knowledge of the president's tweets my able able to profit from them (page 15, line 351). I think such comments are better left out from a scientific paper as to avoid possible attacks that would lower the scientific value of the conclusions. | Thanks for raising this issue (Reviewer 1 also has brought it up – comment #3). We realize that we should have toned down as it might distract from the argumentation. We amended the choice of words in the text fragments mentioned in this comment. {65-66, 196-197, 360}  We deleted the remark about prior knowledge. {415-416} |
| 20 | page 4, line 106: "the may" -> "may" | ‘they’ {110} |

1. The numbers between {123} refer to the line numbers in the revised version with track changes to indicate the changes in the text in relation to the comment.
