# Supplementary material for: Under his thumb the effect of president Donald Trump’s Twitter messages on the US stock market
Source: PLoS One. 2020 Mar 11;15(3):e0229931. doi: 10.1371/journal.pone.0229931 (PMC7065837; doi:10.1371/journal.pone.0229931)
Supplement: Attachment — Submitted filename: PONE-D-19-26269R1 Rebuttal.docx [file pone.0229931.s003.docx]

PONE-D-19-26269R1

Dear Editor,

We very much appreciate the opportunity to revise and resubmit our manuscript to PLOS ONE. We want to thank you and the reviewers for taking the time to review the manuscript and to provide thoughtful and constructive comments. We address each of them and provide detail below. The line numbers in the response to the comments refers to the document with track changes.

Thank you very much,

Sincerely,

| # | Comment | Response |
| --- | --- | --- |
| Editor | | |
| 1 | Thank you for submitting your manuscript to PLOS ONE. After careful consideration, we feel that it has merit but does not fully meet PLOS ONE’s publication criteria as it currently stands. Therefore, we invite you to submit a revised version of the manuscript that addresses the points raised during the review process. | Thank you very much. We address the points below and hope you feel this results in a manuscript that complies with the publication criteria. |
| 2 | In addition to the points raised by the reviewers, we also ask you to better describe how the sentiment analysis (SentiStrength) works and how could one use it the reproduce the results. | Thanks for raising this, we provide more detail about the sentiment analysis in the main text (lines 264-273) and in the supplementary material (S.A). We did not include this in the main text though as we felt it would break the flow of the analysis. |
| 3 | We also ask you to clearly acknowledge the limitations of observational studies and causality analysis in the abstract and in the main text. | We discuss the limitations of the study in the abstract (lines 23-25) and in the main text (lines 148-150). |
| 4 | A data availability statement also needs to be added in the manuscript (see <https://journals.plos.org/plosone/s/data-availability>). | We include a data availability statement (lines 454-457) and include an appendix with essential information about the events (Supplementary Material F – Event list ). |
| Reviewer #1 | | |
| 5 | I appreciate the replies and modifications the authors provided in response to the comments both by me and by the other reviewer, which I believe have made the manuscript more readable and easy to understand. I still have two comments which I think need to be addressed before the paper is sound for publication. | Thank you very much. We feel your comments have been very helpful. |
| 6 | 1) While I agree with the authors on the choice of only two days in their event window to avoid confounding events and I think 100 events are sufficient to draw some conclusions, I also think that showing some robustness to sample selection would reinforce the results, given the significance of the tests is not particularly strong. I think the authors didn't respond appropriately to the main issue presented by reviewer #2 in their comment #11 (whom I thank for raising the issue which I didn't notice at first), where it was asked a reason for the choice of limiting their sample to the first two years of presidency instead of considering tweets up to present day. Unless the authors have a sound justification for this choice, I would suggest they perform their analysis on the updated dataset. | Thanks for raising this issue. Reviewer #2 had the same question and (s)he was ok with our response (we assume that our responses to the reviewers’ comments are available to both reviewers). An important reason we cannot expand right now is the lack of funding. We have no research budget to take on the additional highly demanding investigation of the tweets. We want to pursue research on this topic and at a later stage compare the impact during several relevant subperiod of the presidency (e.g. first versus second half; first term versus second term, relate to the business cycle). But such comparison requires more observations which are not available yet. We hope that this paper also helps us position well in the competition for research funding which we require to do the additional research which proves very time intensive. |
| 7 | 2) At line 250 (page 10) in the revised manuscript I believe w should be the number of stocks with positive CAR, not negative, in order to be consistent with the statements above. | Thanks for pointing this out, you are correct (see also Campbell et al., 2010, page 3081). We amended the text (lines 257-258). |

| Reviewer #2 | | |
| --- | --- | --- |
| 8 | This is the second version of the paper and I see that all my comments have been considered and taken action upon, as far as it was possible, given the available data. I also appreciate very much the extension of the work taking into consideration the actual numerical strength of the sentiments provided by SentiStrength. I think this add value to the paper.  I still have a few minor comments that I would like you to address before the paper could be published: | Thank you very much. We feel your comments have been very helpful. |
| 9 | 1) You introduce the use of the S&P 500 at page 4. I believe most readers will know what it is. Yet, given its importance in your analysis, a few lines of introduction could be useful to those that have poor knowledge about this index. | Thanks for highlighting this. In order to avoid confusion, we now refer to the stock market index in general on page 4 (lines 93-94). We provide more detail about the S&P500 in the (lines 193-194). |
| 10 | 2) Please re-write your first hypothesis reported at the end of page 5. It is not clear. | We rewrote the first hypothesis (lines 125-126). |
| 11 | 3) I really appreciate your clear explanation of what an "event study" is, at page 6. It is really clear now and also justify the small window of time you considered. | Thank you very much. We are pleased that you feel the event study is clearly explained and motivated. |
| 12 | 4) You should motive more clearly some of the paper's decision, like for example the one related to removing companies involved in mergers and acquisitions (page 7). Why? The president could have also commented on them too! Also, at page 11, you (still) did not justify the use of the Jarque-Bera statistics for a normal distribution. Why this specific statistics and not, for example the Kolmogorov–Smirnov or the Lilliefors tests, which are better known? | Thanks for raising this issue. We provide an explanation as to why we control for specific confounding news (lines 168-171).  Further, we admit that we don’t have a strong justification for using a specific test to judge the normality of the distribution. Therefore, we decided to leave out the JB-statistic and its probability value (see also comment #14 below) but instead directly refer to the skewness and kurtosis (lines 244-246; 294). |
| 13 | 5) At page 11, Mike Thelwall surname is misspelled at line 276. In general, please double check the text as you might have added some weird english with your editing (e.g. page 18, like 397-398) | We look into this and amended accordingly (e.g., line 264).  We are not sure what you mean by weird English on page 18 (line 397-398) as this is a reference to an article. We did not change the title of the articles in the reference list. |
| 14 | 6) Page 13, you added a line to table 1 with the p-value of the Jarque-Bera statistics. Is 0 correct? Explain it. | Based on your remark regarding the use of tests for normality, we decided to leave out the JB stat and the probability value (see remark #12 above). |
| 15 | 7) Page 19, you removed a sentence on future research on the effects in time of the president tweets. I thought that was very interesting. Why removing it? Also, the very last sentence of the paper reads odd. Written in that way, in my view, it seems to say that you could not establish any lasting effects, while what you wanted to say is that you could not find any experimental support to say that there were lasting effects, although there could have been. The two things are very different and you might want to measure clearly your words when you discuss such "political" topics. | We include the future research on the effects in time (lines 392-394).  We left out the last sentence (lines 399-400) as we do not want to discuss politics. |
|  |  |  |
